# Development of an in vitro multi-enzyme system for efficient one-pot biosynthesis of sorbitol from fructose-6-phosphate

**DOI:** 10.1186/s40643-025-00943-z

**Published:** 2025-09-26

**Authors:** Kai Shen, Chao-Nan Zhu, Jian-He Xu, Gao-Wei Zheng, Qi Chen

**Affiliations:** State Key Laboratory of Bioreactor Engineering, Shanghai Collaborative Innovation Center for Biomanufacturing, East China University of Science and Technology, Shanghai, 200237 China

**Keywords:** Sorbitol-6-phosphate dehydrogenase, Sorbitol-6-phosphate dephosphatase, Fructose-6-phosphate, Multi-enzymatic system, Protein engineering, Sorbitol

## Abstract

Fructose-6-phosphate is a key ketose monophosphate intermediate that plays a central role in the metabolic pathway of glycolysis. Sorbitol is a naturally occurring sugar alcohol with diverse biological properties, which is widely applied in the food, cosmetic, and pharmaceutical industries due to its favorable physicochemical and physiological characteristics. Although a biosynthetic pathway from starch to fructose-6-phosphate has been well established in metabolic engineering studies, the downstream enzymatic route leading to the biosynthesis of sorbitol from this intermediate has not yet been elucidated in the literature. In this study, we performed the design and implementation of an artificial multi-enzymatic cascade system for the biosynthesis of sorbitol from fructose-6-phosphate. Firstly, candidate enzymes sorbitol-6-phosphate dehydrogenase (S6PDH) and sorbitol-6-phosphate dephosphatase (S6PDP) were selected with an initial activity of 147 U/mg and 1.7 U/mg, respectively. Secondly, molecular modifications were then carried out to improve the thermostability of *Ec*S6PDH, *Ec*S6PDH-M4 displays markedly improved thermostability, with a significant extension of half-life at 40 °C from less than 1 min to 375 min and an elevated *T*_m_ by 9.1 °C. Although multiple rounds of protein engineering were undertaken to improve the catalytic activity of *Ec*S6PDP, no variant exhibiting substantially enhanced catalytic efficiency was identified. Finally, a one-pot biosynthetic system was established by integrating *Ec*S6PDH-M4, *Ec*S6PDP, and glucose dehydrogenase. Following systematic optimization of this multi-enzyme cascade system, 82.6 mM of sorbitol was efficiently produced from 200 mM fructose-6-phosphate. Our work provides a foundational framework for the further enzymatic synthesis of sorbitol directly from starch, offering a promising route for sustainable sugar alcohol production.

**Supplementary Information:**

The online version contains supplementary material available at 10.1186/s40643-025-00943-z.

## Introduction

Sorbitol, also known as D-glucitol, is a naturally occurring six-carbon sugar alcohol found in various fruits, such as cherries and apples. It is the most widely used polyol in industrial applications, holding the largest market share among sugar alcohols. Sorbitol is extensively utilized across multiple sectors, including the food, pharmaceutical, cosmetics industries. Notably, approximately 15% of global sorbitol is dedicated to synthesize vitamin C (Gallezot et al. 1994). Sorbitol serves not only as an end product but also as a valuable platform chemical, capable of being converted into key petrochemical derivatives (Li et al. 2009). Furthermore, under alkaline hydrothermal conditions, it can be transformed intolactic acid a versatile intermediate in chemical and biochemical processes (Ramírez-Loṕ et al. 2011).

Currently, sorbitol is mainly prepared by hydrogenation of glucose, and this method has been applied on industry scale (Moreno et al. 2020). In addition to chemical methods, there are many methods of microbial fermentation to produce sorbitol. *Zymomonas mobilis* is an organism that contains glucose-fructose oxidoreductase (GFOR), which can oxidize glucose to produce gluconate-δ-lactone (and then rapidly converted to gluconic acid), and meanwhile reduce the fructose to sorbitol (Liu et al. 2010). A recombinant *Zymomonas mobilis* with high expression of GFOR was constructed, exhibiting at least two-fold higher specific enzyme activity compared to the wild-type strain. Via adding divalent metal ions, the sorbitol yield was improved from 89% to nearly 100%. In parallel, *Lactobacillus casei* has also been explored as alternative platforms for sorbitol biosynthesis. *L. casei* naturally possesses the capability to produce sorbitol under certain conditions. It has been reported that downregulation of lactate dehydrogenase (LDH) activity can effectively reduce lactate formation, thereby minimizing carbon loss during fermentation (Ladero et al. 2007). As *gutB* and *mtlD* genes have been demonstrated to redirect carbon flux towards sorbitol accumulation (Nissen et al. 2005), L. *casei* achieved a lactose-to-sorbitol conversion rate of up to 9.4% through knockdown of these key genes combined with fed-batch fermentation (De Boeck et al. 2010). By employing photosynthetic cyanobacteria as chassis organisms, and implementing the co-expression of sorbitol-6-phosphate dehydrogenase (S6PDH) with fructose-1,6-bisphosphatase, sorbitol production was significantly enhanced in a short-term culture. The final yield of sorbitol was 312 mg/L within 360 h (Chin et al. 2018).

**Fig. 1:**
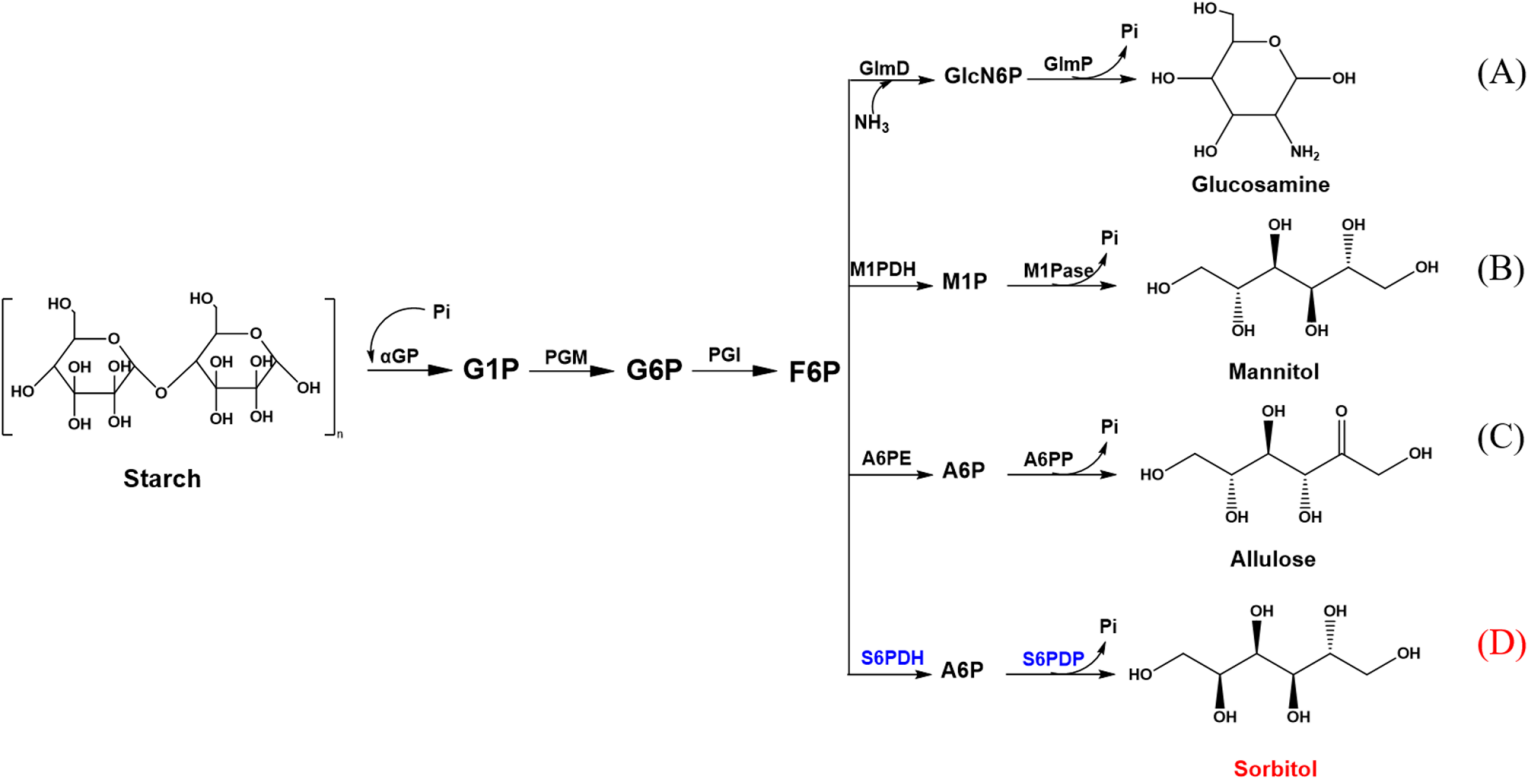
*In vitr*o multi-enzyme catalytic pathways for the synthesis of glucosamine **(A)**, mannitol **(B)**, and allulose **(C)**, along with the designed pathway for sorbitol production **(D)** presented in this study. G1P: glucose-1-phosphate; G6P: glucose-6-phosphate; F6P: fructose-6-phosphate; A6P: allulose-6-phosphate; GlcN6P: glucosamine-6-phosphate; M1P: mannitol-6-phosphate; S6P: sorbitol-6-phosphate. αGP: alpha-glucan phosphorylase; PGM: phosphoglucomutase, PGI: phosphoglucose isomerase; A6PE: allulose-6-phosphate epimerase; A6PP: allulose-6-phosphate phosphatase; GlmD: glucosamine-6-phosphate deaminase; GlmP: glucosamine-6-phosphate phosphatase; M1PDH: mannitol-1-phosphate dehydrogenase; M1Pase: mannitol-1-phosphate phosphatase; S6PDH: sorbitol-6-phosphate dehydrogenase; S6PDP: sorbitol-6-phosphate dephosphatase

In vitro enzymatic methods have emerged as powerful tools for the synthesis of various functional compounds. Recently, several studies have reported the successful in vitro enzymatic production of functional sugars from starch, including allulose (Li et al. 2021), glucosamine (Meng et al. 2020), and mannitol (Wei et al. 2021) (Fig. 1). Notably, when the substrate loading was set at 50 g/L, the corresponding yields reached 79.3%, 47.4%, and 98%, respectively. These pioneering studies inspired us to develop an in vitro enzymatic system for the biosynthesis of sorbitol. In this work, candidate enzymes were systematically screened and engineered. A cascade reaction system was then designed and optimized for the conversion of fructose-6-phosphate to sorbitol. Based on these efforts an efficient biosynthetic pathway from fructose-6-phosphate to sorbitol was successfully established, which provides a feasible approach toward establishing a complete enzymatic pathway for the efficient conversion of starch into sorbitol.

## Materials and methods

### Chemicals and materials

Fructose-6-phosphate was purchased from Hangzhou Diante Biotechnology Co., Ltd, sorbitol was purchased from Shanghai Boer Chemical Reagent Co., Ltd. Trytone and yeast extract were purchased from Oxoid (Hampshire, UK). PrimerSTAR max DNA polymerase was obtained from TaKaRa Biotechnology Co. (Dalian, China). All other chemicals of analytical grade or higher purity were obtained from commercial sources without further purification. All the genes were synthesized by BeijingTsingke Biotech Co., Ltd. *Ch*S6PDP was provide by Tianjin Institute of Industrial Biotechnology, Chinese Academy of Sciences. Glucose degydrogenase from *Bacillus megaterium* (*Bm*GDH) were obtained from the enzyme library deposited in our laboratory.

### Protein expression and purification

The genes encoding *Ec*S6PDH, *Ec*S6PDP were ligated into pET-28a and transformed into *E. coli* BL21 (DE3) for expression. Transformants and glycerin strains were cultured at 37 °C until OD_600_ reached 0.6–0.8 in TB liquid medium with 50 µg/mL kanamycin for pET-28a. Protein expression was induced by isopropyl-β-D-thiogalactopyranoside (IPTG) with a final concentration of 0.2 mM, and the temperature was reduced to 16 °C and incubated at 220 rpm for 24 h. Cells were harvested by centrifuged (10,000 rpm) at 4 °C for 5 min, and resuspended in Tris-HCl buffer (10 mM, pH 7.5). Cell lysates were obtained by cell disruptor and then cell debris was removed by centrifugation at 10,000 rpm for 40 min. The supernatant was added to the nickel column, and the nickel column was rinsed with gradient concentration of Tris-HCl to obtain purified enzyme.

### Rational design of EcS6PDH

FireProt (https://loschmidt.chemi.muni.cz/fireprot/) was used to predict the dominate mutant that could enhance thermostability. Upon inputting the amino acid sequence of *Ec*S6PDH, the system computes the change in folding free energy (ΔΔG) associated with the mutation of one amino acid to another. Following computational analysis, amino acid substitutions are selected for subsequent site-directed mutagenesis based on the criteria that the predicted ΔΔG by FoldX is less than − 1 and the ΔΔG predicted by Rosetta is less than − 2.

### Molecular dynamics simulation of EcS6PDH

Using the LEaP module in the Amber11 software package, the missing hydrogen atoms in the protein-substrate complex of the simulated system were added. The *Ec*S6PDH enzyme was parameterized with the Amber FF19SB force field (Table S1). The complexes were solvated in a cubic box of TIP3P water molecules, with a 12 Å water layer extending outward around the *Ec*S6PDH-fructose-6-phosphate complex, employing periodic boundary conditions.

For the molecular dynamics (MD) simulations conducted using NAMD, energy minimization was first performed for each system, and the system was gradually heated from 0 K to 300 K under the NVT canonical ensemble. After reaching 300 K, the system was equilibrated for 2 ns under the NPT ensemble. Following equilibration, a 100 ns MD simulation was carried out. The van der Waals interactions were gradually eliminated by the switch function, and the long-range electrostatic interactions were calculated using the particle mesh Ewald method with 1.0 Å as the grid parameter.

### Construction and screening of mutation library

Random mutagenesis was conducted by error-prone PCR. The total volume of the target gene fragment reaction system is 50 µL, comprising 2.5 µL of Mn^2+^, 1 µL of template DNA, 3 µL of primers mix (1.5 µL each), 1 µL of rTaq DNA polymerase 4 µL of dNTPs, 5 µL of 10 × PCR buffer and 1 µL of DMSO, made up to 50 µL with water. The total volume of the linearized carrier reaction system is 50 µL, comprising 1 µL of template DNA, 3 µL of primers mix (1.5 µL each), 25 µL of PrimeSTAR max polymerase, and 1 µL of DMSO, made up to 50 µL with water.

PCR amplification protocol for target gene fragments: 95 °C for 3 min; (95 °C for 30 s, 60 °C for 30 s, 72 °C for 50 s) × 30 cycles; 72 °C for 8 min. PCR amplification protocol for linearized carrier: 98 °C for 1 min; (98 °C for 10 s, 60 °C for 15 s, 72 °C for 5.5 min) × 30 cycles; 72 °C for 10 min. The PCR products were resolved by agarose gel electrophoresis and purified. Digestion reaction program: 5 µL of rCutSmart buffer and 2 µL of Dpn I were added for every 50 µL of identified PCR product, and the mixture was incubated for 6 h at 37 °C. Plasmids containing the mutated gene were directly transformed into *E. coli* BL21 (DE3) and then plated on a Luria–Bertani (LB) agar plate with 100 µg/mL of ampicillin. Directly transform the plasmid containing the mutated gene into *E. coli* BL21 (DE3), and then plate it with 50 µg/mL kanamycin on Luria Bertani (LB) agar plates. Pick the bacteria from the agar plate and place them in a first level 96 well plate containing 50 µg/mL kanamycin, incubate at 37 °C for 12 h, then transfer the bacterial cells from the first level plate to a second level 96 well plate containing 50 µg/mL kanamycin, incubate at 37 °C for 3.5 h, induce with 0.1 mM isopropyl-β-D-thioiogalactopyranoside (IPTG), and incubate at 16 ° C for 24 h.

Use lysozyme to crush the collected bacterial cells. Divide the crude enzyme solution into two parts and dilute it to an appropriate concentration. One part is stored at 4 °C, and the other part is incubated at 50 °C for a certain period of time before being cooled at 4 °C for 10 min. Add 1 mM fructose-6-phosphate and 0.15 mM NADH to Tris-HCl buffer (200 mM, pH 7.5) and mix well. Add 150 µL of mixed buffer to each well of the enzyme-linked immunosorbent assay (ELISA) plate, and then add 50 µL of crude enzyme solution to each well after insulation, quickly place it in the ELISA reader to measure the change in absorbance at 340 nm for 1 min, calculate the residual activity of the crude enzyme after insulation, and select the best one for rescreening. The dominant mutants obtained from the initial screening were cultured in 250 mL shake flasks, and the collected bacteria were crushed and purified to obtain pure enzymes.

After diluting the purified enzymes of each mutant, they were packaged and incubated at 45 °C for a period of time. After cooling in an ice bath for 5 min, their activity was measured using a spectrophotometer, and their residual activity was calculated and compared.

### Enzyme assay

The specific activities of S6PDH were measured by monitoring the initial change of the absorbance of NADH cofactors at 340 nm (e = 6220 M^− 1^cm^− 1^). The specific activities of S6PDP were measured by the change of inorganic phosphorus concentration at A850 (Saheki et al. 1985). For S6PDH, reactions were performed in a 1 mL mixture, comprising 970 µL Tris-HCl buffer (200 mM, pH 7.5), 10 µL fructose-6-phosphate (3 mM), 10 µL NADH (0.15 mM) and an appropriate amount of S6PDH. For S6PDP, reactions were performed in a 400 µL mixture, comprising 350 µL Tris-HCl buffer (200 mM, pH 7.5), 10 µL fructose-6-phosphate (1 mM), 10 µL NADH (1 mM), 10 µL Mg^2+^ (10 mM), 10 µL excessive *Ec*S6PDH, and an appropriate amount of S6PDP.

### Optimization of multi-enzyme cascade reaction conditions

The optimization parameters included temperature, pH, addition ratios of *Ec*S6PDH, *Bm*GDH and *Ec*S6PDP, fructose-6-phosphate/glucose addition ratios, NAD^+^ concentration, and Mg^2+^ concentration. Temperature conditions were tested at 25 °C, 30 °C, 35 °C and 40 °C; pH conditions were measured from 6.0 to 6.5 (100 mM citric acid-sodium citrate buffer) and 7.0-8.5 (200 mM Tris-HCl buffer); enzyme addition ratios were 2:2:0.5, 2:2:1, 2:2:2, 2:2:2.5, 2:2:3 and 2:2:4; fructose-6-phosphate/glucose addition ratios were set to 1:0.5, 1:1, 1:1.5, 1:2 and 1:2.5, respectively; NAD^+^ were measured from 0.05 to 0.25 mM, and the addition of Mg^2+^ was 0–30 mM.

### Analytical methods

Quantitative analysis of sorbitol using high pressure liquid chromatography (HPLC). The chromatographic column used was Bio-Rad AminexHPX-87 H, with a mobile phase of 5 mM sulfuric acid, a flow rate of 0.5 mL/min, and a column temperature of 40 °C. The detector used was a differential refractive index detector.

### One pot multi-enzymatic cascade system

The one-pot reaction mixture (1 mL) contained 20 mM fructose-6-phosphate, 0.1 mM NAD^+^, 10 mM Mg^2+^, 10 mM glucose, 2 U/mL *Ec*S6PDH, 2 U/mL *Bm*GDH and 1 U/mL *Ec*S6PDP. The reaction mixtures were shaken at 35 °C and 1000 rpm for 1 h. To terminate the reaction, it was placed in a metal bath at 80 °C for 10 min.

### High substrate loading reaction

A high substrate loading mixture (10 mL), containing 200 mM Tris-HCl buffer (pH 8.0), 200 mM fructose-6- phosphate, 0.15 mM NAD^+^, 15 mM Mg^2+^, 300 mM glucose, 0.2 U/mL *Ec*S6PDH, 0.2 U/mL *Bm*GDH and 0.25 U/mL *Ec*S6PDP. Magnetic stirring of the reaction system at 35 °C for 30 h, and 6 M NaOH was used to regulate pH. Samples were taken at intervals and placed in an 80 °C metal bath to terminate the reaction.

## Results and discussion

### Construction of a dual-enzymatic cascade system

In order to construct a dual-enzymatic cascade system for synthesizing sorbitol, the candidate enzymes of sorbitol-6-phosphate dehydrogenase (S6PDH) and sorbitol-6-phosphate dephosphatase (S6PDP) were identified that could catalyze the conversion of fructose-6-phosphate to sorbitol-6-phosphate and the conversion of sorbitol-6-phosphate to sorbitol. *Aa*S6PDH from *A. arboris* (Liss et al. 1962), *Ec*S6PDH from *E. coli* K12 (Céline et al. 2006), *Ea*S6PDH from *E. amylovora* (Salomone-Stagni et al. 2018) were selected. The specific activities of these enzymes were determined (shown in Table 1) and *Ec*S6PDH with the highest specific activity (147 ± 2.5 U/mg) was finally selected.

**Table 1:** Comparative of specific activity of S6PDH from diverse origins

Entry	Enzyme	Gene	Organism	Specific activity (U/mg)
1	*Aa*S6PDH	srlD	*A. arboris*	117 ± 1.1
2	*Ec*S6PDH	srlD	*E. coli* K12	147 ± 2.5
3	*Ea*S6PDH	srlD	*E. amylovora*	4.3 ± 0.7

Reaction conditions: 1 mL of the mixture contains 970 µL Tris-HCl buffer (200 mM, pH 7.5), 10 µL fructose-6-phosphate (3 mM), 10 µL NADH (0.15 mM) and an appropriate amount of S6PDH.

For sorbitol-6-phosphate dephosphatase (S6PDP), Pyp1 from yeast (Xu et al. 2018), *Ec*S6PDP and *Ec*S6PDP1 from *E. coli* K12 (Taejun et al. 2018), *Ch*S6PDP was selected. The specific activities of these enzymes were determined (shown in Table 2) and *Ec*S6PDP with the highest specific activity (1.7 ± 0.2 U/mg) was selected.

**Table 2:** Comparative of specific activity of S6PDP from diverse origins

Entry	Enzyme	Gene	Organism	Specific activity (U/mg)
1	Pyp1	YNL010W	Yeast	n.d. ^a^
2	*Ec*S6PDP	YfbT	*E. coli* K12	1.7 ± 0.2
3	*Ec*S6PDP1	YidA	*E. coli* K12	n.d. ^a^
4	*Ch*S6PDP	nagD	*C. hutchinsonii*	0.8 ± 0.1

Reaction conditions: 400 µL of the mixture contains 350 µL Tris-HCl buffer (200 mM, pH 7.5), 10 µL fructose-6-phosphate (1 mM), 10 µL NADH (1 mM), 10 µL Mg^2+^ (10 mM), 10 µL excessive *Ec*S6PDH, and an appropriate amount of S6PDP.

^a^ not detected.

To evaluate the capability of the selected enzymes to synthesize sorbitol, *Ec*S6PDH and *Ec*S6PDP were employed in a one-pot enzymatic system. Upon the addition of 3 mM fructose-6-phosphate (F6P), the reaction was carried out for 12 h at 30 °C. The results indicated that the product yield reached 3 mM, achieving a conversion efficiency of 100%. High pressure liquid chromatography (HPLC) and mass spectrometry were employed to confirm the identity of the product as sorbitol (Fig. S1, Fig. S2). Both analytical methods verified the successful production of sorbitol.

### Protein engineering to improve the thermostability of EcS6PDH

To address the unsatisfactory thermostability of *Ec*S6PDH (Table 4), a combination of rational design and error-prone PCR strategies were employed to carry out multiple rounds of thermostability enhancement. Firstly, the online prediction tool FireProt was employed to identify the potential mutants that could enhance the thermostability of *Ec*S6PDH. This analysis led to the selection of nine mutants that met the predefined criteria (Table 3). Single-point mutations and combinational mutations were introduced at these nine identified positions. Experimental results identified the double mutant Q66D/G74M (designated as M1), which showed enhanced thermostability. M1 displayed a half-life of 31 min at 40 °C, a significant improvement compared to the wild-type enzyme. Meanwhile, the specific activity of M1 was increased by 9.5% compared to the wild-type (Table 4).

**Table 3:** Potential mutants estimated using fireprot

Position	Reference	Alter	FoldX	Rosetta
66	Q	D	-1.1	-5.2
74	G	M	-2.2	-2.2
104	D	M	-1.3	-2.6
106	D	I	-1.1	-2.1
152	S	W	-2.1	-2.2
186	N	Y	-1.4	-2.2
217	D	L	-1.5	-2.9
225	C	I	-1.3	-4.1
245	C	Y	-1.1	-2.3

Following the rational design of *Ec*S6PDH, three rounds of random mutagenesis were carried out, leading to the screening of a total of 15,000 mutants. Finally, M4 (Q66D/G74M/A92V/A23VM233V/A40E) was obtained, exhibiting a significant improvement in thermostability with the half-life at 40 °C increasing from less than 1 min to 375 min and the melting temperature *T*_m_ rising from 46.2 °C to 55.3 °C (Table 4). However, the specific activity of M4 was slightly reduced compared to the wild-type. Nevertheless, considering that its enzymatic activity remains at a relatively high level, the current thermostability and catalytic efficiency of M4 are sufficient for practical applications.

**Table 4:** Characterization of the specific activity and thermostability of sorbitol-6-phosphate dehydrogenase

Enzyme	Position						Specific activity (U/mg)	t_1/2_ (40 °C, min)	T_m_ (°C)
	66	74	92	23	233	40			
**WT**	Q	G	V	A	M	A	147 ± 2.3	< 1	46.2 ± 0.2
**M1**	D	M	V	A	M	A	161 ± 2.1	31	47.4 ± 0.1
**M2**	D	M	A	A	M	A	133 ± 2.7	84	49.7 ± 0.1
**M3**	D	M	A	V	V	A	160 ± 1.7	305	53.9 ± 0.2
**M4**	D	M	A	V	V	E	138 ± 1.6	375	55.3 ± 0.1

### Mechanism analysis of thermostability improvement of EcS6PDH

To gain insights into the structural basis of the enhanced thermostability exhibited by the *Ec*S6PDH mutant M4, molecular dynamics simulations were carried out for both the wild-type *Ec*S6PDH (*Ec*S6PDH-WT) and the M4 mutant (*Ec*S6PDH-M4) (Fig. S3). According to the Root Mean Square Fluctuation (RMSF) analysis, the overall structural fluctuations of the mutant *Ec*S6PDH-M4 system did not exhibit significant differences compared to the wild-type. However, a reduction in flexibility was observed primarily at amino acid positions 145–155 and 180–200 (Fig. S4).

**Fig. 2:**
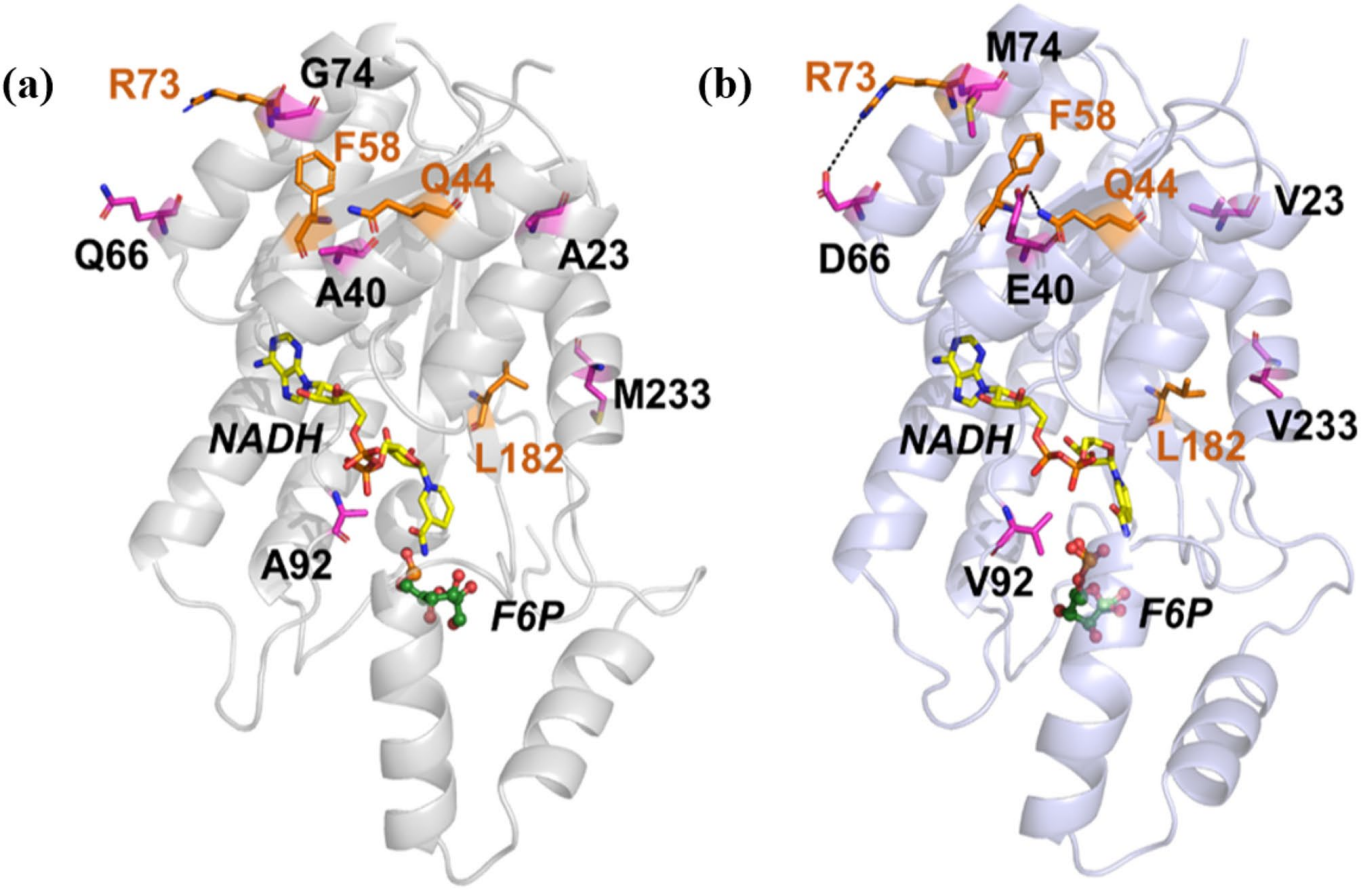
Interactions between *Ec*S6PDH and surrounding amino acids before **(a)** and after **(b)** mutation

The changes in interactions between the mutation sites and surrounding amino acids before and after mutation were analyzed in each system (Fig. 2). The results revealed that mutations in certain amino acids introduced additional hydrophobic interactions, such as V23, M74, V92, and V233. In addition, other amino acids substitutions led to the formation of novel stabilizing interactions. For example, the mutation of Q66 to D66 resulted in a stable electrostatic interaction between D66 and R73, while the replacement of A40 to E40 facilitated the formation of a new hydrogen bond with the side chain of Q44. These newly established interactions are postulated to contribute to the overall structural stability of *Ec*S6PDH-M4, thereby enhancing its thermostability.

### Construction and optimization of the tri-enzyme cascade system

To address the issue of the low catalytic activity of the *Ec*S6PDP enzyme, a semi-rational design approach based on structure–function relationship analysis was undertaken. 23 amino acid residues within a 4 Å around the substrate, as well as 15 residues lining the substrate access channel were selected as target sites for mutagenesis. Amino acid scanning strategy using A, S, F, and Y was carried out at these positions. However, no mutant with significantly improved specific activity was identified. This may be attributed to the limited library size screened due to the lack of a high-throughput screening method.

**Scheme 1:**
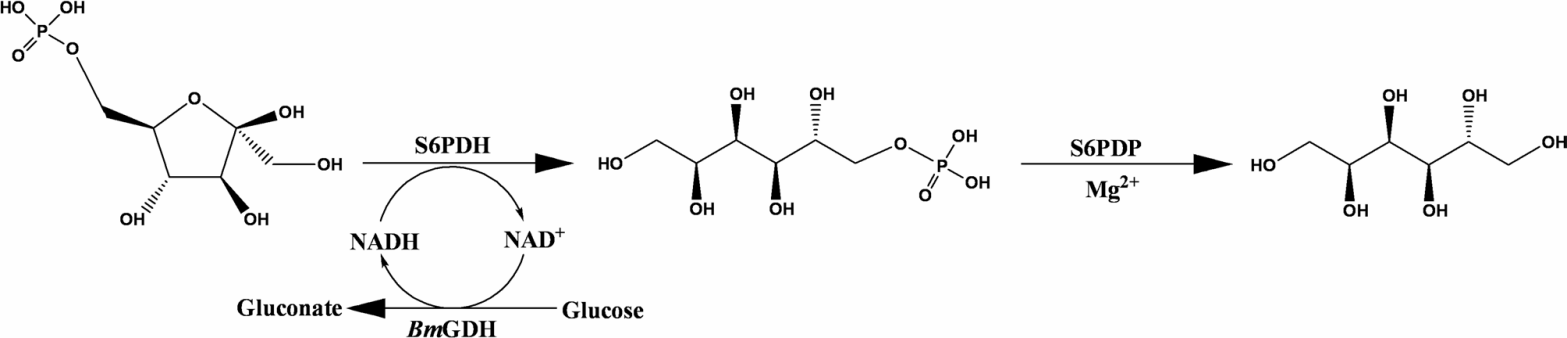
Tri-enzymatic cascade for the synthesis of sorbitol from fructose-6-phosphate

Finally, by integrating the thermostable variants *Ec*S6PDH-M4, the *Ec*S6PDP selected through the screening, and a glucose dehydrogenase *Bm*GDH for NADH regeneration, the tri-enzymatic cascade system was constructed to enable the biosynthesis of sorbitol from fructose-6-phosphate as starting substrates (Scheme 1).

**Fig. 3:**
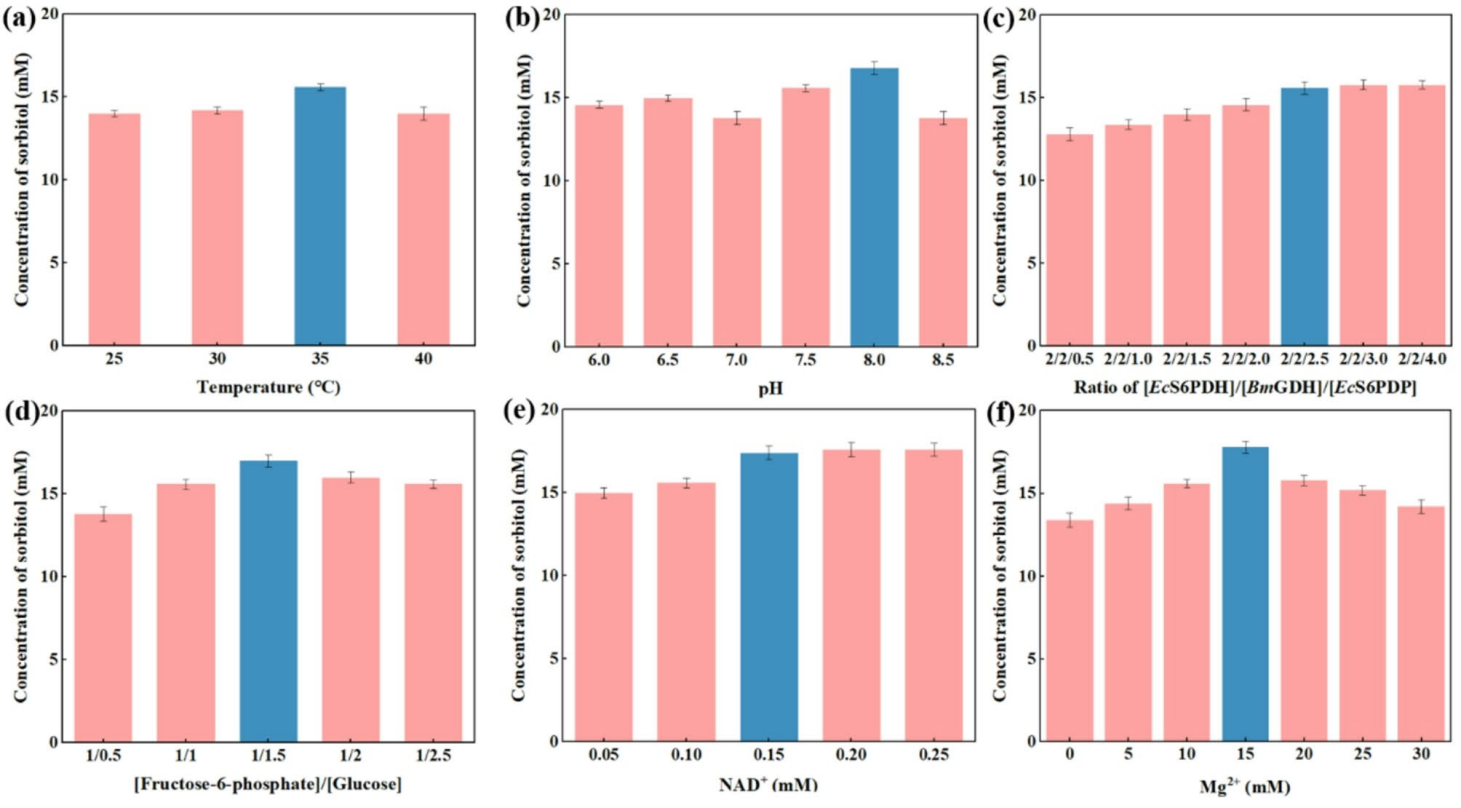
Optimization of the temperature **(a)**, pH **(b)**, ratio of [*Ec*S6PDH]/[*Bm*GDH]/[*Ec*S6PDP] **(c)**, dose ratio of [Fructose-6-phosphate]/[Glucose] **(d)**, dose of NAD^+^**(e)** and dose of Mg^2+^**(f)**

We further optimized the reaction conditions of the system to overcome the compatibility issues among the distinct enzymatic catalysts within the multi-enzyme cascade system. The optimization included adjusting parameters such as reaction temperature, pH, enzyme ratios [*Ec*S6PDH]/[*Bm*GDH]/[*Ec*S6PDP], ratio of [Fructose-6-phosphate]/[Glucose], NAD^+^ concentration, and MgCl_2_ concentration. Finally, the optimal reaction condition was determined as the optimal temperature was 35 °C and optimal pH was 8.0 (Fig. 3a, b), the optimal ratio of [*Ec*S6PDH]/[*Bm*GDH]/[*Ec*S6PDP] and [Fructose-6-phosphate]/[Glucose] for were 2/2/2.5 and 1/1.5 (Fig. 3c, d), and the dose of NAD^+^ and Mg^2+^ were 0.15 mM and 15 mM respectively (Fig. 3e, f).

**Fig. 4:**
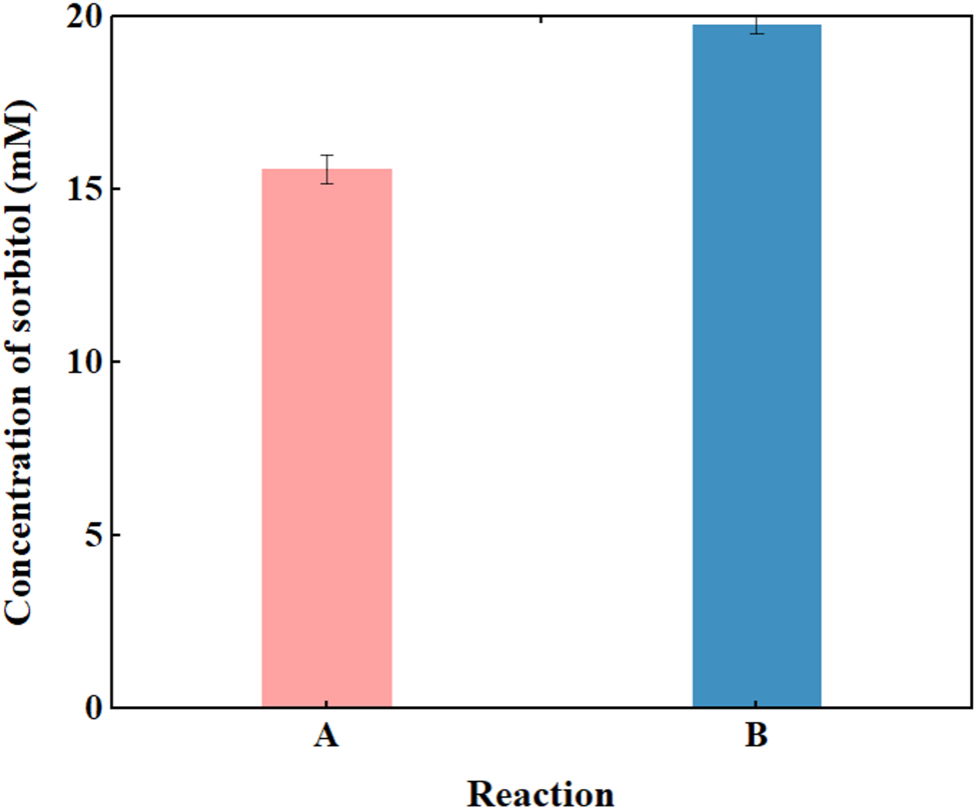
Sorbitol concentration before and after reaction optimization. Before optimization **(A)** and after optimization **(B)**. The reaction condition before optimization: 20 mM fructose-6-phosphate, 2 U/mL *Ec*S6PDH, [*Ec*S6PDH]/[*Bm*GDH]/[ *Ec*S6PDP] = 2/2/1, 0.10 mM NAD^+^, 20 mM glucose, 10 mM Mg^2+^, Tris-HCl buffer (200 mM, pH 7.5), 35 °C, 1 h, 1000 rpm; The reaction condition after optimization: 20 mM fructose-6-phosphate, 2 U/mL *Ec*S6PDH, [*Ec*S6PDH]/[*Bm*GDH]/[ *Ec*S6PDP] = 2/2/2.5, 0.15 mM NAD^+^, 30 mM glucose, 15 mM Mg^2+^, Tris-HCl buffer (200 mM, pH 8.0), 35 °C, 1 h, 1000 rpm

The final concentration of product sorbitol before and after the reaction optimization was compared as shown in Fig. 4. When the substrate fructose-6-phosphate concentration was set 20 mM, the final sorbitol concentration prior to reaction optimization was 15.6 mM. Following optimization, and the sorbitol concentration increased to 19.8 mM within 1 h, which is close to the theoretical maximum under these conditions.

### Scaled-up multi-enzyme cascade reaction

**Fig. 5:**
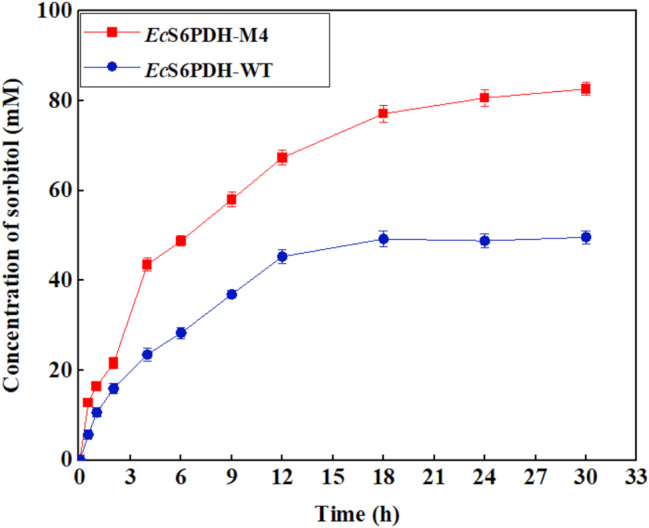
Biosynthesis of sorbitol with high substrate loading. Reaction conditions: 10 mL of the mixture contains 200 mM F6P, 300 mM glucose, 0.15 mM NAD^+^, 15 mM Mg^2+^, 0.2 U/mL *Ec*S6PDH-WT/*Ec*S6PDH-M4, 0.2 U/mL *Bm*GDH, 0.25 U/mL *Ec*S6PDP, 200 mM Tris-HCl buffer (pH 8.0), 35 °C

Furthermore, an attempt was made to expand the reaction scale to examine the reaction effect: increasing the substrate addition to 200 mM and reducing the enzyme activity addition of *Ec*S6PDH to 0.2 U/mL, other conditions remain unchanged. 10 mL of reactions were carried out and samples were taken at different time step during the 30 h reaction (Fig. 5). When adding the thermostable mutant *Ec*S6PDH-M4, the final concentration of sorbitol can reach 82.6 mM (15 g/L), while the final concentration of sorbitol is only 49.6 mM (9 g/L) when *Ec*S6PDH-WT is added. This result demonstrated that the tri-enzyme reaction system constructed through protein engineering and reaction optimization exhibited high catalytic efficiency in the conversion of fructose-6-phosphate to sorbitol.

## Conclusions

In summary, by recruiting sorbitol-6-phosphate dehydrogenase, sorbitol-6-phosphate dephosphatase and glucose dehydrogenase, an efficient and convenient multi-enzyme cascade reaction was successfully constructed to product sorbitol from fructose-6-phosphate. After optimization of the reaction, 200 mM loaded fructose-6-phosphate was smoothly converted into 82.9 mM sorbitol (15 g/L) in 30 h. Our work provides a theoretical basis for the industrial production of sorbitol.

More works of protein engineering may be necessary to improve the catalytic activity of sorbitol-6-phosphate dephosphatase, thus further increasing the yield of sorbitol. This multi-enzyme cascade system provides a certain foundation for in vitro multi enzyme catalysis of starch conversion to sorbitol.

## Supplementary Information

Below is the link to the electronic supplementary material.

Supplementary Material 1

Supplementary Material 2

## Data Availability

The datasets used and analyzed during the current study are available from the corresponding author on reasonable request.
