# Supplementary material for: Development of an in vitro multi-enzyme system for efficient one-pot biosynthesis of sorbitol from fructose-6-phosphate
Source: Bioresour Bioprocess. 2025 Sep 26;12(1):104. doi: 10.1186/s40643-025-00943-z (PMC12474828; doi:10.1186/s40643-025-00943-z)
Supplement: Supplementary file 1 — Supplementary Material 1 [file 40643_2025_943_MOESM1_ESM.docx]

**Supporting information**

**Development of an *In Vitro* Multi-Enzyme System for Efficient One-Pot Biosynthesis of Sorbitol from Fructose-6-Phosphate**

Kai Shen^1^，Chao-Nan Zhu^1^，Jian-He Xu^1^，Gao-Wei Zheng^1^*，Qi Chen^1^*

^1^ State Key Laboratory of Bioreactor Engineering and Shanghai Collaborative Innovation Center for Biomanufacturing, East China University of Science and Technology, Shanghai 200237, China.

* Corresponding authors: Gao-Wei Zheng, gaoweizheng@ecust.edu.cn; Qi Chen, chenqi_angela@163.com


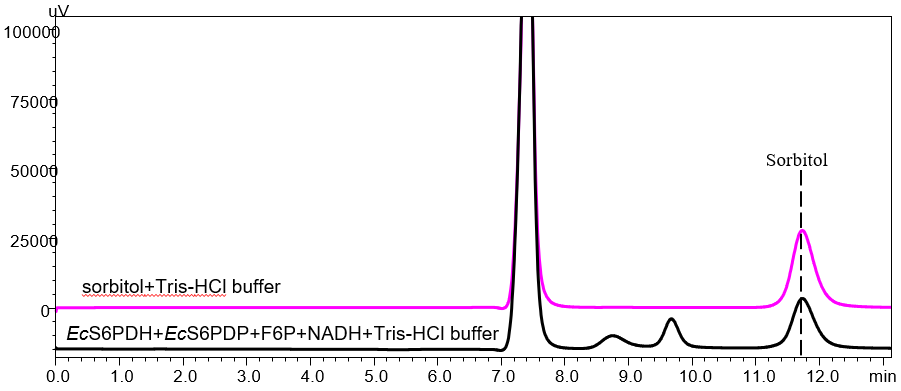


**Figure S1.** Identification of products by HPLC. Standard sorbitol (pink), reaction solution (black).


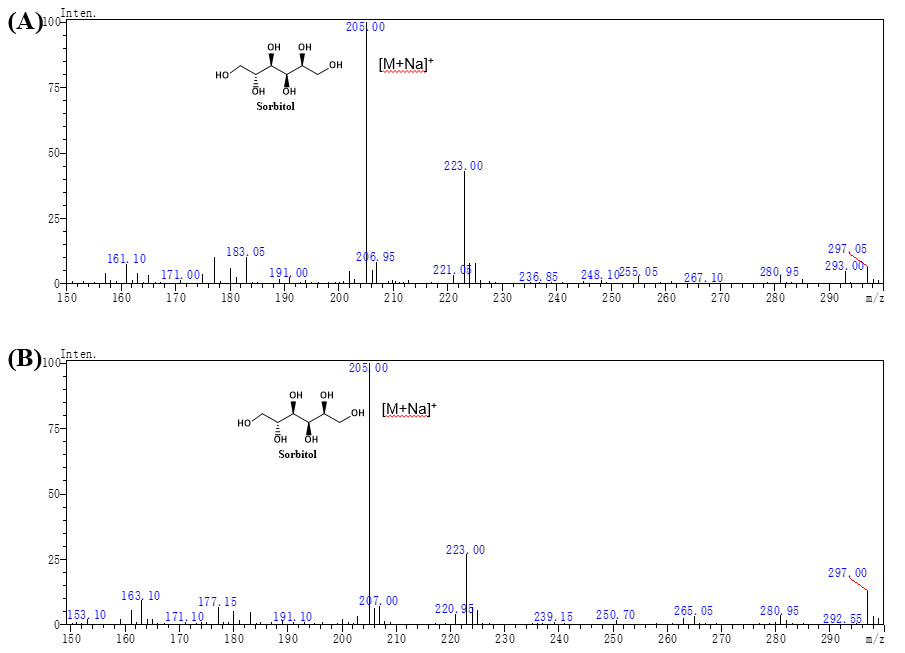


**Figure S2.** MS analysis of dual enzyme cascade reaction from F6P to sorbitol. Standard sorbitol (**A**) and reaction solution (**B**).

**Table S1.** Partial charges of *Ec*S6PDH. (a) Partial charges of *Ec*S6PDH, (b) Bond, angle and torsion parameters of *Ec*S6PDH.

**Table S1.** (a) Partial charges of *Ec*S6PDH

| **Index** | **Name** | **Coordinate**  **x** | **Coordinate**  **y** | **Coordinate**  **z** | **Type** |  |  | **Partial**  **charges** |
| --- | --- | --- | --- | --- | --- | --- | --- | --- |
| 1 | C1 | 0.58 | -0.932 | -0.867 | c3 | 1 | LIG | 0.132473 |
| 2 | C2 | 1.813 | -1.519 | -0.177 | c3 | 1 | LIG | 0.222354 |
| 3 | H1 | 2.548 | 1.204 | -1.737 | ho | 1 | LIG | 0.412037 |
| 4 | H2 | 3.225 | -0.738 | -1.225 | ho | 1 | LIG | 0.394055 |
| 5 | H3 | 1.974 | -1.751 | 2.369 | ho | 1 | LIG | 0.366549 |
| 6 | C3 | 1.411 | 0.855 | 0.461 | c3 | 1 | LIG | 0.614763 |
| 7 | H4 | -1.299 | 1.375 | 0.646 | ho | 1 | LIG | 0.489499 |
| 8 | C4 | 2.106 | -0.497 | 0.929 | c3 | 1 | LIG | 0.126146 |
| 9 | C5 | -0.734 | -1.292 | -0.179 | c3 | 1 | LIG | 0.03807 |
| 10 | C6 | 2.476 | 1.878 | 0.012 | c3 | 1 | LIG | 0.137144 |
| 11 | O1 | -1.799 | -0.736 | -0.884 | os | 1 | LIG | -0.52819 |
| 12 | O2 | 0.845 | 0.438 | -0.857 | os | 1 | LIG | -0.53246 |
| 13 | O3 | 0.565 | 1.36 | 1.28 | o | 1 | LIG | -0.85969 |
| 14 | O4 | 1.607 | -0.899 | 2.181 | oh | 1 | LIG | -0.64533 |
| 15 | O5 | 2.91 | -1.622 | -1.069 | oh | 1 | LIG | -0.74068 |
| 16 | O6 | 3.228 | 1.46 | -1.122 | oh | 1 | LIG | -0.71411 |
| 17 | P1 | -2.965 | 0.109 | -0.069 | p5 | 1 | LIG | 1.372905 |
| 18 | O7 | -4.024 | 0.41 | -1.053 | o | 1 | LIG | -0.8714 |
| 19 | O8 | -3.258 | -0.646 | 1.174 | o | 1 | LIG | -0.8714 |
| 20 | O9 | -2.18 | 1.477 | 0.265 | oh | 1 | LIG | -0.78154 |
| 21 | H5 | 0.534 | -1.261 | -1.901 | h1 | 1 | LIG | 0.058028 |
| 22 | H6 | 1.654 | -2.521 | 0.214 | h1 | 1 | LIG | 0.031984 |
| 23 | H7 | 3.186 | -0.369 | 1.003 | h1 | 1 | LIG | 0.00448 |
| 24 | H8 | -0.717 | -0.968 | 0.851 | h1 | 1 | LIG | 0.05507 |
| 25 | H9 | -0.834 | -2.381 | -0.177 | h1 | 1 | LIG | 0.05507 |
| 26 | H10 | 3.18 | 2.067 | 0.815 | h1 | 1 | LIG | 0.017078 |
| 27 | H11 | 1.95 | 2.801 | -0.212 | h1 | 1 | LIG | 0.017078 |

**Table S1.** (b) Angle parameters of *Ec*S6PDH

| **ANGLE** | **K_θ_ (kcal/mol**  **e/radian^2^)** | **θ (*degrees*)** |  |
| --- | --- | --- | --- |
| o-c3-os | 74.4 | 122.3 | same as o-c3-o , penalty score = 5.5 |

**
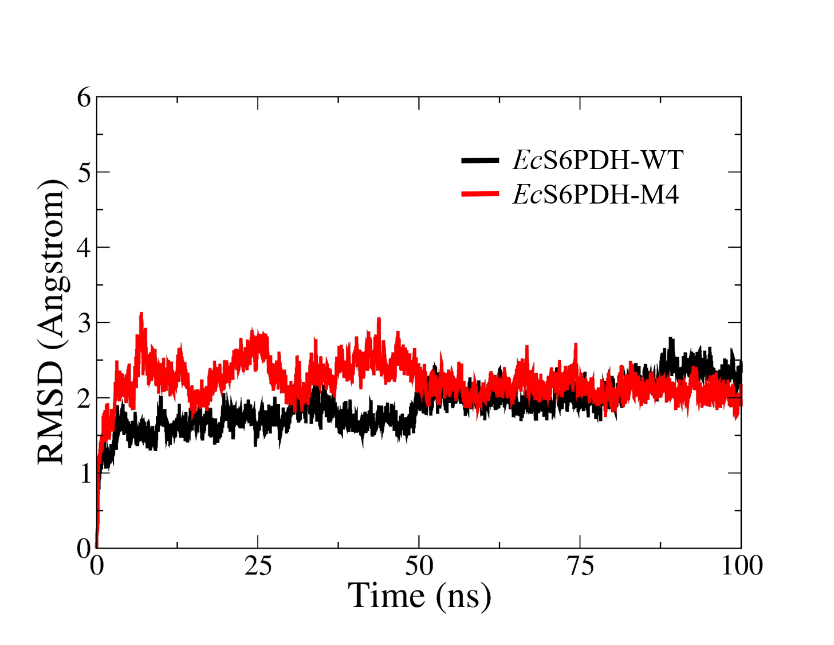
**

**Figure S3.** RMSD calculation results of *Ec*S6PDH-WT and *Ec*S6PDH-M4.

**
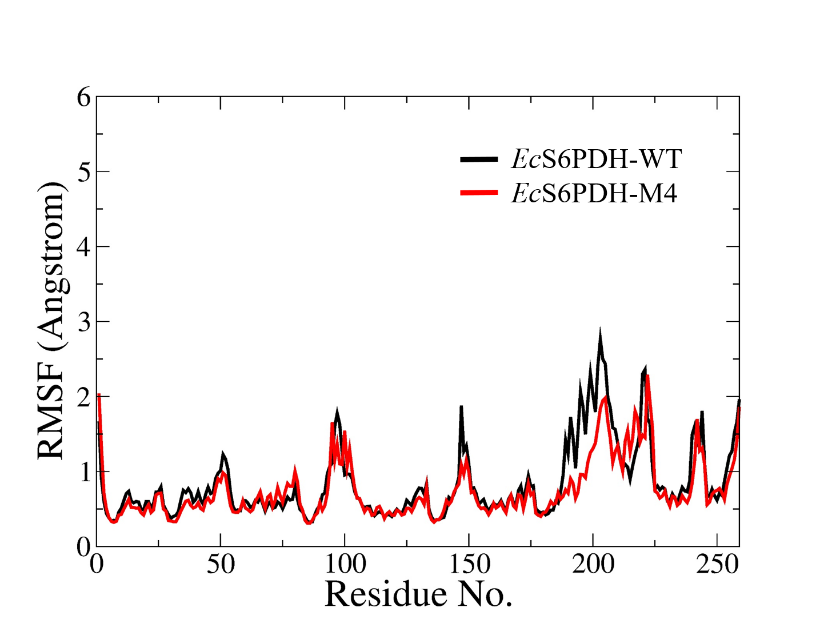
**

**Figure S4.** RMSF calculation results of *Ec*S6PDH-WT and *Ec*S6PDH-M4.
