# Supplementary figures and images for: Development of an in vitro multi-enzyme system for efficient one-pot biosynthesis of sorbitol from fructose-6-phosphate
Source: Bioresour Bioprocess. 2025 Sep 26;12(1):104. doi: 10.1186/s40643-025-00943-z (PMC12474828; doi:10.1186/s40643-025-00943-z)

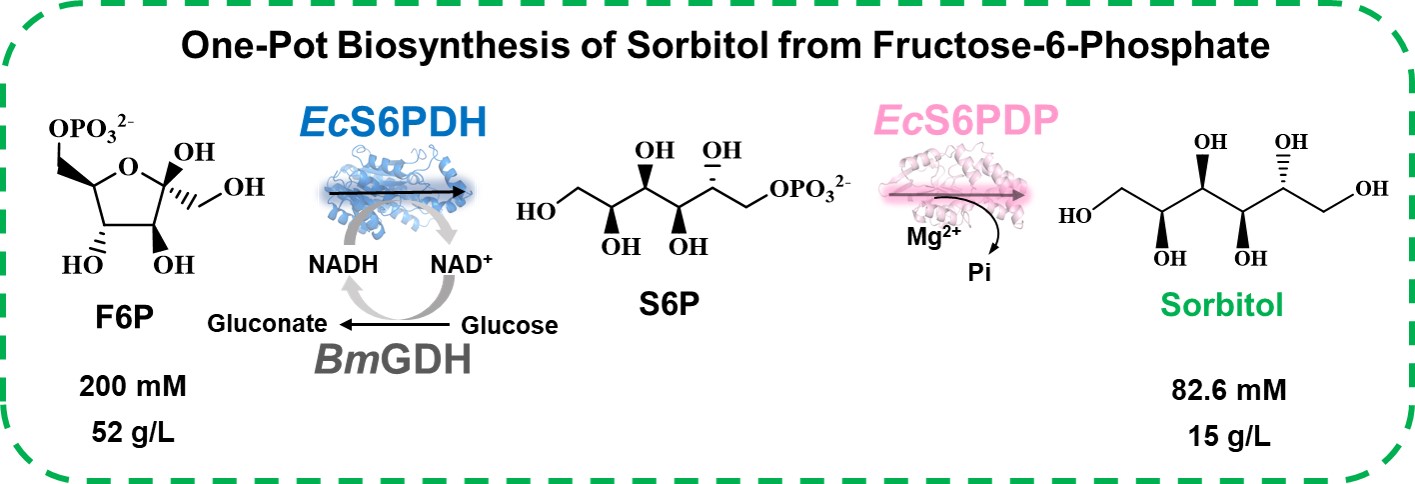

Supplement: Supplementary file 2 — Supplementary Material 2 [file 40643_2025_943_MOESM2_ESM.jpg]
